# Zebrafish Embryo Developmental Toxicity Assay (ZEDTA) for Regulatory Testing—Protocol Optimization and Historical Control Data

**DOI:** 10.3390/toxics13100874

**Published:** 2025-10-14

**Authors:** Daphne van den Oetelaar, Marysia Agnieszka Tobor-Kapłon, Mèlanie Reijnaers, Manon Beekhuijzen

**Affiliations:** 1 Developmental and Reproductive Toxicology, Charles River Laboratories, 5231 DD ’s-Hertogenbosch, The Netherlands; daphne.vandenoetelaar@crl.com (D.v.d.O.); manon.beekhuijzen@crl.com (M.B.); 2 Environmental Toxicology, Charles River Laboratories, 5231 DD ’s-Hertogenbosch, The Netherlands; 3 Syngenta Crop Protection BV, 4611 AP Bergen op Zoom, The Netherlands; marysia.tobor-kaplon@syngenta.com

**Keywords:** zebrafish, zebrafish embryo developmental toxicity assay (ZEDTA), optimization, teratogenicity, historical control data

## Abstract

The Zebrafish Embryo Developmental Toxicity Assay (ZEDTA) is a promising and innovative method with potential to replace the screening of teratogenic substances in mammals during preclinical development. However, a harmonized and validated protocol does not exist for the ZEDTA, and data on the background incidence of spontaneous malformations are not readily accessible. Therefore, the aim of this research was twofold: (1) to optimize the ZEDTA protocol and (2) to generate historical control data. The most optimal results were achieved by exposing zebrafish larvae in 24-well plates at a temperature of 26 °C in combination with the renewal of test solutions after 48 h of exposure. Furthermore, the use of 0.5% *v*/*v* DMSO did not induce more malformations or mortality than exposure to standard ISO medium. In total, 26 valid experiments were conducted using the optimized ZEDTA protocol. An overall mortality of 3.5% was recorded after 96 h of exposure. Malformations were observed in 7.6% of all surviving larvae. The most frequently observed abnormalities included yolk sac deformation (4.0%), followed by tail (2.8%), heart (2.6%), and head malformations (1.6%). The optimized protocol was considered effective in supporting an optimal development rate of exposed zebrafish larvae, with low mortality and minimal background malformations. These findings indicate a low level of confounding factors and high reliability of results, making an essential step in the refinement of ZEDTA toward global harmonization and regulatory acceptance.

## 1. Introduction

Regulatory developmental toxicity assessments for pharmaceuticals, agrochemicals, and industrial chemicals are traditionally conducted using mammalian models, primarily rats and rabbits [1,2,3]. However, the ongoing commitment to the 3R principles—Reduction, Refinement, and Replacement of animal testing—has triggered the development of innovative in vitro alternatives. One alternative could be the Zebrafish Embryo Developmental Toxicity Assay (ZEDTA) [4,5,6].

According to the EU Directive 2010/63/EU, zebrafish embryos are not classified as laboratory animals until they are capable of independent feeding, which generally begins within the first five days post-fertilization [7,8]. As the ZEDTA is conducted within this non-protected period of five days, it presents a significant opportunity to reduce the use of vertebrate animals in developmental toxicity testing. Additionally, the optical transparency of zebrafish embryos throughout early development enables continuous monitoring of the development of the whole organism from early embryogenesis until hatching and beyond (early larval stages) [9]. Compared to conventional in vivo developmental toxicity studies in rats and rabbits, the ZEDTA not only aligns with the 3R principles but also offers practical advantages, being lower in costs and faster. Zebrafish are easy to maintain, produce large numbers of offspring per week, and undergo rapid development. Additionally, Zebrafish embryos are already applied in regulatory toxicology through the Fish Embryo Acute Toxicity Test (FET; OECD Test Guideline 236) [10], which is accepted in the EU for acute toxicity assessment. While the FET does not address teratogenicity, its regulatory status illustrates the feasibility of embryo-based assays and provides a precedent for the potential adoption of the ZEDTA.

In order to implement the ZEDTA for regulatory testing, further refinement and standardization of the test protocol are required. There have already been several initiatives aimed at optimizing the ZEDTA [11,12,13,14], and Beekhuijzen et al. [15] took the first steps towards harmonization of the protocol by proposing a set of optimal test conditions based on methods described in the literature. Despite these advancements, as also indicated by Beekhuijzen et al. [15], additional characterization and validation of these assay conditions are still required to enable regulatory adoption. A particular challenge has been the lack of comprehensive information on spontaneous malformations, which is essential for defining optimal test conditions and for the reliable interpretation of a normal background level of malformations.

In parallel, Mori et al. [16] strengthened the regulatory relevance of zebrafish assays by developing an atlas of chemically induced malformations under the ICH S5(R3) guideline. Their study confirmed that zebrafish replicate more than 80% of mammalian malformation and embryo–fetal lethality outcomes, supporting translational concordance between systems. Importantly, they also showed that spontaneous malformations are rarely observed when embryos are carefully selected, underscoring the value of well-defined historical controls. Together, these findings complement the ongoing optimization of ZEDTA by providing a regulatory framework for malformation classification and supporting broader international harmonization.

Building on these complementary developments, the present study aimed to (1) develop an optimized ZEDTA protocol with test conditions optimal for embryonic and larval development, while being cost-effective and reproducible under standard laboratory conditions, and (2) to generate a historical control dataset detailing background mortality rates and the incidence of common malformations of zebrafish embryos and larvae under blank control conditions.

## 2. Materials and Methods

The presented research consists of two parts: (1) the optimization of the ZEDTA protocol and (2) the collection of background data (i.e., data from the blank control treatment) obtained from 26 experiments conducted using the optimized procedure.

### 2.1. General Study Design

The general outline of the ZEDTA was based on the OECD technical guideline No. 236: “Fish Embryo Acute Toxicity (FET) Test” [10]. Fertilized eggs of zebrafish wildtype Tüebingen were obtained from Zodiac, Wageningen UR, NL, or from an in-house culture (adult fish of the same line were also obtained from Zodiac). The adult zebrafish were housed in a ZebTEC system, with pH (7.5), conductivity (700), and temperature (25 °C) automatically adjusted. Pre-selection of eggs was performed using an Olympus CK2 ULWCD 0.3 light microscope. Only fertilized eggs with a round chorion and no signs of coagulation were selected. Embryos were selected during their blastula phase (2–4 h post-fertilization (hpf)) and incubated in a climatic chamber (Micro Clima Economic Lux, Snijders Labs, Tilburg, The Netherlands). Light intensity was generally between 550 and 1080 lux and a 14 h light period per day was applied. Plates were covered with self-adhesive film to prevent evaporation.

Exposure medium was prepared using RO water (tap water purified by reverse osmosis; GEON Waterbehandeling, Berkel-Enschot, The Netherlands) and contained CaCl_2_·2H_2_O at 211.5 mg/L, MgSO_4_·7H_2_O at 88.8 mg/L, NaHCO_3_ at 46.7 mg/L, and KCl at 4.2 mg/L. Initial oxygen concentration was ≥80% of the saturation value, and pH ranged between 6.5 and 8.5. The temperature was variable depending on the test.

**Figure 1:**
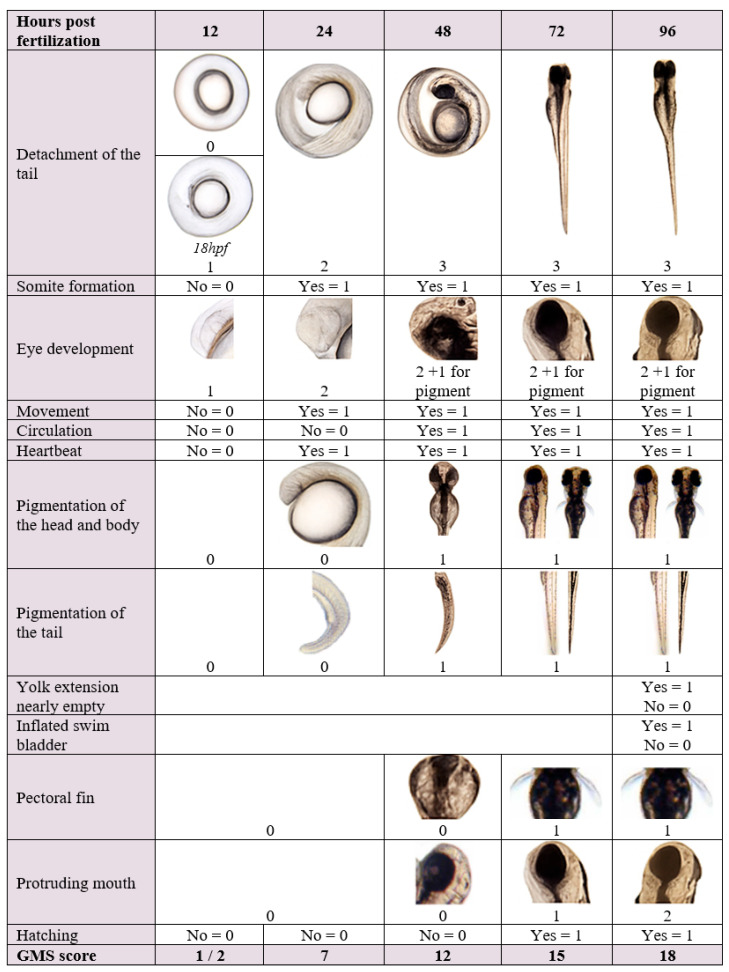
Extended General Morphology Score (Extended GMS). Two corrections were made to the extended GMS scoring system published by Beekhuijzen et al. [15]: (1) At 24 h after exposure, a heartbeat can be observed but no circulation is visible. (2) At 96 h after exposure, an additional observation was included: an inflated swim bladder (yes = 1, no = 0). Consequently, the maximum score at this time point increased to 18 points.

Observations of hatching, survival, and general development using the extended General Morphology Score (extended GMS, see Figure 1) [15] were performed at 24 h intervals, i.e., at 24, 48, 72, and 96 h following the start of exposure (i.e., 28, 52, 76, and 100 hpf). Dead embryos/larvae were recorded and removed when observed.

At the end of exposure, the developmental (Figure 1) and teratogenic endpoints (see Table 1) were assessed. Endpoints that required observations on live larvae were assessed first (i.e., movement, pectoral fins, and teratogenicity of the head and body). The teratogenic endpoints were scored as either present (score 1) or absent (score 0). Larvae were then euthanized with 1.2% ethylene glycol monophenylether. Afterwards, the larvae were assessed further for remaining developmental and teratogenic endpoints (i.e., eye development, swim bladder, and yolk sac).

OECD guideline 236, on which these experiments were based, requires that a valid test should comply with the following criteria: hatching rate in the control should be ≥80% at the end of 96 h exposure, and the post-hatch survival in the control should be ≥90% until the end of the 96 h exposure. Based on this, the results of a plate were considered valid and used for further analysis when overall survival was ≥80% (i.e., cumulative pre- and post-hatching survival).

### 2.2. Optimization of Study Protocol

The experimental conditions that were tested were based on Beekhuijzen et al. [15] and consisted of a comparison of (1) the use of a solvent, (2) different incubation temperatures, (3) different refreshment periods, and (4) different exposure volumes. The following exposure conditions and their combinations were tested:

1. Use of solvent (0.5% *v*/*v* DMSO vs. blank exposure medium). In the experiments in which the effects of DMSO were tested, a volume of the blank medium was spiked with DMSO (Merck, Darmstadt, Germany) at a loading of 5 mL per liter medium and subsequently distributed to test vessels. In total, 1081 zebrafish embryos/larvae were tested in a blank control (47 plates) and 241 embryos/larvae with a solvent control (12 plates), with different combinations of the remaining parameters in valid experiments.
2. Temperature (26 vs. 28 °C). In total, 842 embryos/larvae (38 plates) were tested at 26 °C and 480 embryos/larvae (21 plates) at 28 °C, with different combinations of the remaining parameters in valid experiments.
3. Renewal periods (static vs. semi-static). For the semi-static exposure, during the renewal of test solutions, the old medium was removed using a Pasteur pipette either every 24 h or once after 48 h of exposure. Each time a small volume of medium was left to prevent exposure of the embryo/larvae to air. Fresh medium, pre-heated to the desired temperature, was added with a pipette. Care was taken not to touch the embryo/larvae. In total, 930 embryos/larvae were exposed in a 24 h semi-static design (42 plates), 48 embryos/larvae in a 48 h semi-static design (7 plates), and 231 embryos/larvae in a static design (10 plates), with different combinations of the remaining parameters in valid experiments.
4. Exposure vessels (24-well vs. 96-well plates). Twenty-four embryos/larvae were exposed in 24-well plates; each well contained 2.0 mL of medium. Twenty embryos/larvae were exposed in 96-well plates; each well contained 0.2 mL of medium. One embryo/larva was exposed per well. In total, 1143 embryos/larvae were tested on 49 “24-well” plates and 179 embryos/larvae on 10 “96-well plates”, with different combinations of the remaining parameters in valid experiments.

#### 2.2.1. Growth

In a number of tests, larval length was measured at the end of the exposure as a measure of growth. Not all larvae from a given plate were measured. To prevent bias, larvae were selected for measurement in a randomized manner. In total, 658 larvae were measured in valid experiments. Different combinations of exposure parameters described as above were tested. The effect of the solvent was not included in this assessment.

#### 2.2.2. Data Evaluation

Plates fulfilling the validity criterion of at least 80% embryonic survival (i.e., pre- and post-hatching survival) were used for further analysis. One plate with 79% survival was also included. A qualitative comparison of the obtained results was performed. No statistical analysis was performed as data interpretation was based not only on biological results but also on ethical and practical considerations.

The following parameters were calculated for each plate separately and, subsequently, an average and standard deviation (for survival) or mode (most frequent value, for development) were calculated for each treatment:

- Survival (%) was calculated as a percentage of the number of larvae surviving the 96 h exposure period divided by the initial number of eggs.
- Effects on development as assessed with the extended GMS were based on the most frequent score (mode) on a given time point per plate.
- Teratogenicity:
  - Individual malformations: For each plate, the percentage of larvae showing a given malformation was calculated.
  - Total score: For each organism, a teratogenic score, i.e., a sum of all malformations, was calculated (integer 0–7; 0: no malformations; 7: seven malformations). Next, the average total score per plate was calculated.

### 2.3. Historical Control Data Obtained with the Optimized Protocol

All eggs used for this part of the study were obtained from Zodiac, Wageningen UR, NL. Control data from twenty-six experiments (i.e., 26 blank control plates), which met the validity criterion of at least 80% survival in the control treatment, were available for analysis. All these experiments were performed under the same conditions (see “general study design”). Based on the optimization part of this project (as described in the former chapter), the following test conditions were used:

- For each experiment, twenty-four embryos in the blastula phase were exposed to the exposure medium on a 24-well plate.
- Four embryos (last column of the well plate) served as internal control and were scored only for survival but not for development and teratogenicity.
- Exposure temperature was 26 ± 1.0 °C, and the medium was renewed once after 48 h.

The length of larvae at the end of the exposure was not measured in these experiments. Survival was calculated for each plate separately and, subsequently, an average was calculated for all experiments together. The percentage of larvae showing a given malformation was calculated for all data together. For each larva, a teratogenic score was calculated and, subsequently, an average was calculated for all zebrafish. For the developmental rate, a distribution of scores was calculated for each time point based on all data.

## 3. Results

### 3.1. Optimization of Study Protocol

#### 3.1.1. Mortality

A total of 80 plates were used in this study. Of these, 60 plates met the predefined validity criterion of ≥80% embryonic survival and were included in the final analysis. Among the 20 invalid plates that did not reach the cut-off criterion, one plate showed a very low survival rate of 4%, whereas the remaining 19 had an average survival rate of 67% (range: 54–75%). Of the 60 valid plates, 59 showed embryonic survival rates of ≥83%, from which 27 plates showed no mortality. On one plate, the survival was 79%, which was still considered acceptable, and this plate was therefore included in the analysis.

Data on survival from valid experiments are presented in Table 2. Among these experiments, the average survival in the blank control was slightly higher, at 95%, compared to the survival in the 0.5% *v*/*v* DMSO control (91%). The effect of temperature on survival was negligible within the blank control treatment: survival rates were 94% at 26 °C and 95% at 28 °C. In 0.5% *v*/*v* DMSO, survival at 26 °C was slightly higher (93%) than at 28 °C (90%).

As for the type of exposure plate, survival was lower in 96-well plates than 24-well plates in both control treatments: for the blank control, survival was 96% in 24-well plates and 90% in 96-well plates, and in the 0.5% *v*/*v* DMSO controls, survival was 93% and 89% in the 24- and 96-well plates, respectively.

In addition to temperature and plate type, the effect of the refreshment interval on mortality was also assessed. In the blank control, survival moderately increased with longer refreshment periods: survival was 93% in 24 h semi-static conditions, 96% in 48 h semi-static conditions, and 97% with no refreshment (static condition) over the 96 h experiment. It should be noted that for the 0.5% *v*/*v* DMSO control, only the 24 h semi-static condition was tested.

Overall, the highest observed mean survival rate (100%) occurred under blank control conditions, in 24-well plates, at 28 °C, and with a static exposure regime.

#### 3.1.2. Development

To assess the effects of the different treatment conditions on larval development, the General Morphology Scores (GMSs) of the larvae were assessed. Table 3 summarizes the modal GMS for each combination of treatments at different exposure time points. Table 4 shows the distribution of the GMSs at the end of the exposure period.

During the first 72 h of exposure, no clear differences in general development were observed between treatments. At 96 h, the end of the experiment, development was retarded in most 96-well plates: not a single larva reached the optimal development stage (GMS 18), except for the condition with 0.5% *v*/*v* DMSO at 26 °C, where 33% of the larvae reached a GMS of 18. Furthermore, static conditioning, i.e., no refreshment during the full exposure period, also delayed the development. This was most pronounced at 26 °C, with no larvae reaching a GMS of 18, while at 28 °C, 33% of larvae reached this optimal developmental stage.

**Scheme 1:**
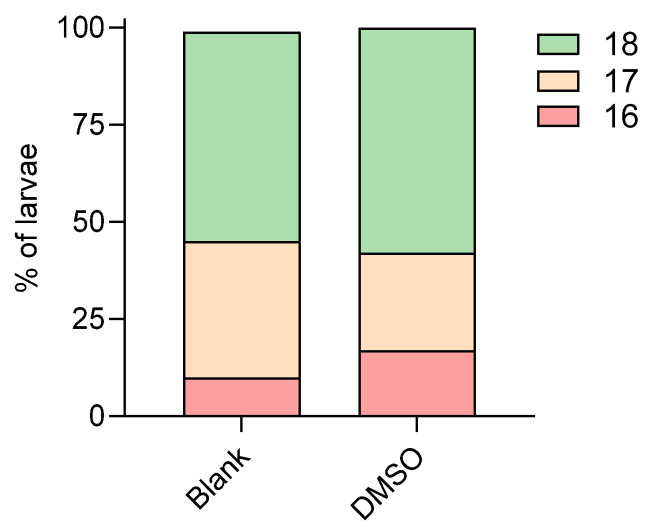
Distribution of developmental scores in blank control and 0.5% *v*/*v* DMSO at the end of the exposure (96 h).

**Scheme 2:**
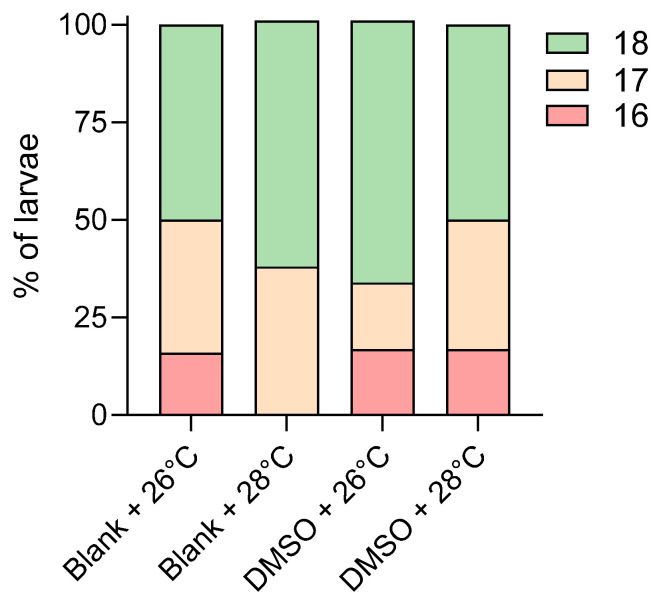
The influence of temperature (26 vs. 28 °C) on the distribution of developmental scores in different solvents at the end of the exposure.

**Scheme 3:**
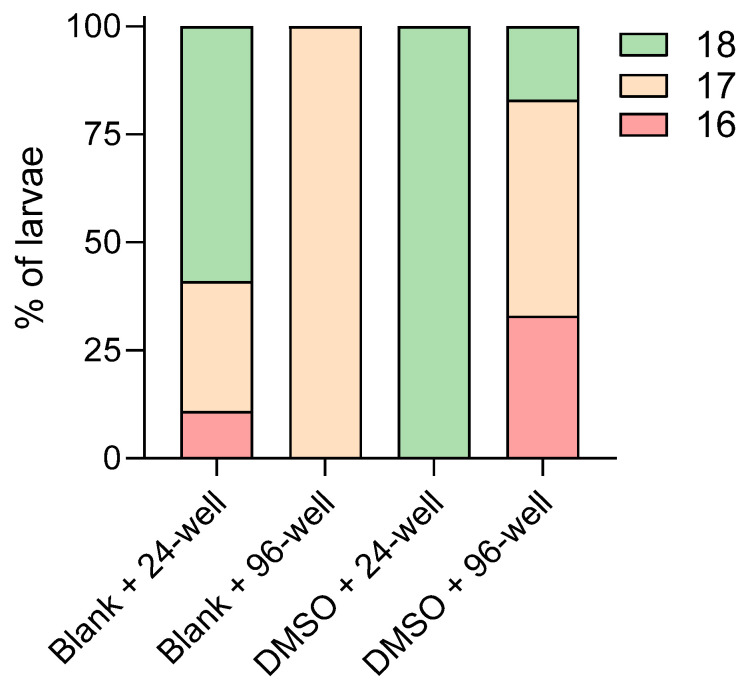
Distribution of developmental scores in different well plates (24- vs. 96-well) at the end of the exposure, grouped by the control treatment (optimization study).

**Scheme 4:**
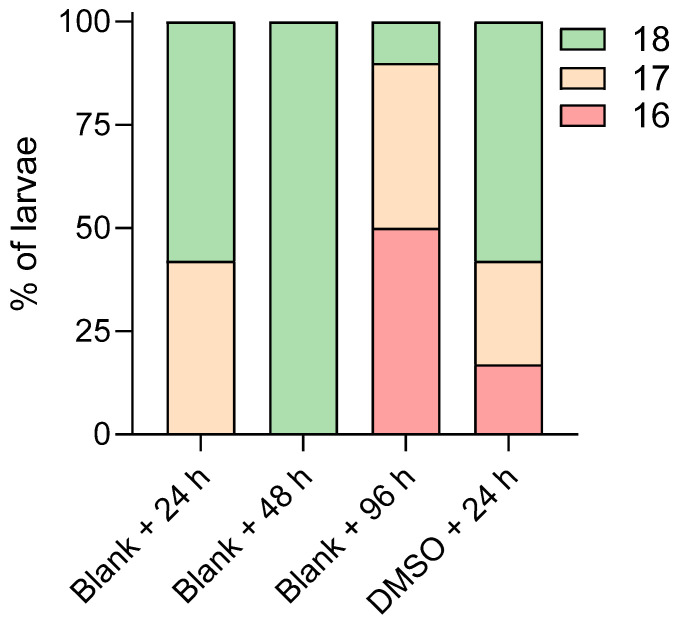
Distribution of developmental scores following different refreshment periods (24, 46, and 96 h) at the end of the exposure, grouped by the control treatment.

The distribution of modal developmental scores was assessed separately for the used solvent, the temperature, the plate type, and the refreshment interval to determine the individual effects of the different treatment conditions (Scheme 1, Scheme 2, Scheme 3 and Scheme 4).

The use of 0.5% *v*/*v* DMSO did not influence the larvae development when compared to the blank control; a similar distribution of developmental scores was observed between both control conditions (Scheme 1).

The effect of temperature on development at the end of exposure was more pronounced in the blank control than in the 0.5% *v*/*v* DMSO control (Scheme 2). In the blank control at 26 °C, 16% of larvae showed a developmental score of 16, a score of 17 was reached by 34% of larvae, and 50% reached a score of 18. A shift towards higher developmental scores was observed in the blank control at 28 °C (37.5% and 62.5%, with scores of 17 and 18, respectively). In the 0.5% *v*/*v* DMSO control, differences between temperatures were less obvious: at 26 °C, 17%, 17%, and 67%, they showed a developmental score of 16, 17, and 18, respectively. At 28 °C, the distribution was 17%, 33%, and 50%, respectively.

For the well-plate size, in the blank control, the use of a 24-well plate appeared beneficial to development over the use of a 96-well plate. For 24-well plates, a half-normal distribution was observed with 11, 30, and 59% of larvae showing a score of 16, 17, and 18, respectively, whereas in 96-well plates, all larvae presented with a score of 17. In the 0.5% *v*/*v* DMSO control, this effect was more pronounced with all larvae exposed in 24-well plates showing a score of 18, while in 96-well plates, the distribution of modal scores among 16, 17, and 18 was 33, 50, and 17%, respectively (Scheme 3).

For the frequency of media refreshment, a refreshment period of 48 h in blank control resulted in the most optimal developmental score for all larvae (Scheme 4). The developmental score was reduced with a refreshment period of 24 h, relatively independent of the use of 0.5% *v*/*v* DMSO as a solvent. Development was most retarded when no media refreshment was performed, with 50% of larvae only reaching a GMS of 16. It should be noted that for the 0.5% *v*/*v* DMSO control, only a refreshment period of 24 h was assessed.

Overall, the most optimal developmental scores were reached in larvae incubated in 24-well plates, with a 48 h refreshment period at 28 °C.

#### 3.1.3. Growth

Table 5 summarizes the effects of different treatment conditions on the length of larvae at the end of exposure. Larvae length was assessed only for the blank control treatment.

There was no apparent difference in the growth of larvae incubated at different incubation temperatures (2.9 ± 1.0 at 26 °C and 3.2 ± 0.91 mm at 28 °C (mean ± standard deviation)). At 26 °C, under a 24 h refreshment period, the average length of larvae incubated in 96-well plates was higher than those incubated in 24-well plates (3.6 ± 0.2 vs. 3.0 ± 1.1 mm, respectively), while no apparent differences were observed at 28 °C (3.5 ± 0.85 vs. 3.4 ± 0.27 mm for 24 and 96 wells, respectively).

The effect of the refreshment schedule on the growth of larvae was more apparent. When static exposure was applied, larvae were approximately half the size of larvae incubated under semi-static renewal (3.3 ± 0.89, 3.6 ± 0.26, and 1.7 ± 0.18 mm for 24, 48, and 96 h refreshment period, respectively).

Overall, the greatest average larval length was observed in larvae incubated at 26 °C in 24-well plates with a 48 h refreshment interval.

#### 3.1.4. Malformations

Surviving larvae were observed for a number of malformations, i.e., malformations of the head, saccule/ortholiths, tail, whole body shape, heart, and yolk abnormalities (Table 6).

Malformation of the heart was the most frequently observed abnormality, followed by yolk sac malformations, occurring in an average of 12% and 8.1% of larvae, respectively. The least frequent malformation was of the sacculi/otholiths (0.23%), observed only in two treatment conditions: 24-well plates with 24 h solution refreshment at both 26 °C and 28 °C. Head malformations were the next least common (1.0%), followed by tail (3.0%) and body shape malformations (3.2%).

Although each treatment condition showed a different pattern of observed malformations, in general, exposure to 0.5% *v*/*v* DMSO resulted in slightly fewer abnormalities compared to the blank control (average teratogenic scores: 0.27 vs. 0.32, respectively). The most obvious difference between these two groups was the frequency of yolk sac malformations: 3.7% of larvae in the 0.5% *v*/*v* DMSO group were affected versus 9.1% in the blank control.

Also, the effect of temperature varied across treatment conditions. In general, in blank controls, lower temperatures were associated with a higher teratogenic score (0.41 at 26 °C vs. 0.23 at 28 °C). In contrast, the 0.5% *v*/*v* DMSO plates showed the opposite trend, with higher scores at 28 °C (0.37) compared to 26 °C (0.17).

Well size significantly influenced malformation frequency. In both control groups, 96-well plates consistently showed higher teratogenic scores than 24-well plates. In the blank control, average scores were 0.56 (96-well) and 0.24 (24-well), while in the 0.5% *v*/*v* DMSO group, scores were 0.43 and 0.11, respectively. Abnormalities of the heart, body shape, and yolk sac also tended to occur more frequently in the smaller wells.

Refreshment frequency had an evident effect on the frequency of malformations. In the blank control, the lowest teratogenic score was obtained for the 48 h refreshment period (0.20), while a static design reached an average score of 0.30.

Overall, the size of the well plates had a stronger effect than the renewal period, as the highest scores for malformations consistently occurred in 96-well plates when compared with 24-well plates. The overall lowest teratogenic scores were observed at 28 °C in 24-well plates with 24–48 h refreshment intervals.

### 3.2. Control Data of Optimized Protocol

Based on the acquired data as described above, considering ethical aspects (see Discussion Section 4), the most optimal conditions for the ZEDTA were determined to be as follows: incubation in a 24-well plate at 26 °C with renewal of medium at 48 h after initiation of exposure.

The blank controls of a new set of experiments conducted at these specific conditions were used for the collection of historical control data on the mortality rate and the incidence of spontaneous malformations in zebrafish embryos and larvae, the results of which are described below.

#### 3.2.1. Mortality

A total of 26 valid experiments were performed using the optimized study setup. In these experiments, a total of 520 embryos were tested in blank medium. The average mortality in all experiments increased from 2.9% after 24 h of exposure and 3.3% after 48 and 72 h of exposure to 3.5% at the end of the exposure (see Table 7, last column). Thus, in total, 18 embryos did not survive the 96 h exposure period. In 15 of the 26 experiments, no mortality was observed. Only one plate showed increased mortality of 20% (4 out of 20 exposed zebrafish).

#### 3.2.2. Development

Table 7 presents the distribution of developmental scores at each time point. At all time points following the start of exposure, the majority of the embryos/larvae scored the maximum or second-highest score of that given time point according to the extended GMS. It was, however, noticeable that the number of larvae reaching the maximum developmental score decreased with longer exposure times: at 24 and 48 h of exposure, more than 85% of embryos reached the maximum developmental score, whereas at the later time points, this was reduced to 60–62%.

#### 3.2.3. Malformations

Under the optimized protocol, no malformations were observed in 11 out of 26 experiments. Abnormalities were found in 38 larvae, which equals 7.6% of all surviving zebrafish (Table 8). The majority of affected larvae showed only one malformation (68%, which is 5.2% of all surviving larvae). In total, 12 larvae showed more than one malformation (i.e., 2.4% of all surviving larvae). One larva showed six and one larva presented with seven malformations. Both were described as totally malformed.

Table 9 provides an overview of the frequency of each type of malformation. The most frequent were yolk malformations (30%; 3.98% of all surviving larvae), tail malformations (21%; 2.79% of all surviving larvae), and heart malformations (20%; 2.59% of all surviving larvae). Eight larvae presented with malformations on the head (12%; 1.59% of all survival larvae), six larvae showed malformations of pectoral fins (9.1%; 1.2% of all surviving larvae), malformation of the body shape was noted in four larvae (6.1%; 0.80% of surviving larvae), and one larva was found with malformations of sacculi/otholiths (1.5%; 0.20% of all surviving larvae).

The relative frequency of malformations observed in this historical control data study was similar to the observations made during the optimization study with the same treatment conditions, where yolk and heart malformations were also the most frequently observed. The average teratogenic score in this study was 0.13, which was lower compared to the same combination of treatments during the optimization phase, i.e., 0.27.

## 4. Discussion

To aid global adoption and harmonization of the ZEDTA for regulatory developmental toxicity assessments, this study had two primary objectives: (1) optimization of the ZEDTA protocol and (2) generation of historical control data under optimized conditions. Test conditions as described in Beekhuijzen et al. [15], including temperature, exposure volumes, refreshment periods, and solvent use, were systematically assessed to establish the most suitable conditions for further testing. The optimized conditions, as established under the first objective, were subsequently used to perform a number of tests which enabled the collection of historical control data, essential to support the interpretation of future study results.

The article of Beekhuijzen et al. [15] recommended, amongst others, the following conditions:

- An incubation temperature of 26 °C.
- A 24-well plate (2 mL per well) combined with self-adhesive film as a test chamber.
- Static, semi-static, and flow-through systems were discussed as exposure methods, but an optimal method was not defined.
- In total, 0.1% *v*/*v* DMSO was considered safe for use, but it was noted that the most optimal would be to perform the assay without any solvent.

Zebrafish tolerate temperatures between 25 and 33 °C, but optimal breeding conditions are considered to be reached between 27 and 28.5 °C [9,17]. It is also known that the rate of development will increase with rising temperature [18]. In the optimization study described in this current paper, a lower incubation temperature of 26 °C resulted in a shift towards lower developmental scores, confirming the aforementioned findings. Furthermore, higher teratogenicity scores were noted at 26 °C compared to incubation at 28 °C. An incubation temperature of 28 °C appears more beneficial for larval development. However, performing the assay at 28 °C may accelerate growth, potentially allowing larvae to reach the protected feeding phase (i.e., independent feeding) before the end of the 96 h assay. This early development could exceed the regulatory threshold, thereby undermining a key advantage of the assay: reduction in the number of animals used in toxicology testing. In addition, it has been shown that the ZEDTA is more sensitive to teratogenic effects at lower temperatures, potentially because the larvae stay in a specific developmental stage that is more sensitive to teratogenicity for a longer period of time at a lower temperature [15]. Overall, we can conclude that 26 °C is the most optimal incubation temperature to promote assay sensitivity while taking ethical considerations into account.

In addition to the effects of temperature, we assessed whether the size of the incubation plate affects the outcomes of the assay. The use of 96-well plates resulted in lower larval survival, overall lower developmental scores, and higher teratogenicity scores when compared with incubation in 24-well plates. Malformations such as heart and yolk malformations, kinked tails, and generally deformed body shapes were noted more often in embryos that were placed in 96-well plates. This is in accordance with observations made by Selderslaghs et al. [19], likely attributable to limited swimming space and possible accumulation of waste. Surprisingly, embryos exposed in 96-well plates at 26 °C were, on average, longer than embryos exposed in 24-well plates at the same temperature. The reason for this cannot be determined by the setup of the current study, but it is possible that under suboptimal conditions (low oxygen supply, low swimming space, and buildup of wastes), energy allocation is moved towards growth instead of proper development [20]. No clear difference in larval length was noted between the two types of well plates when incubated at 28 °C, which might be due to the more favorable growth conditions at this temperature, resulting in relatively higher larval growth in the 24-well plate.

An infrequently investigated parameter in the ZEDTA is the influence of the frequency of media refreshment. Our results showed that the semi-static method resulted in a lower incidence of malformations and a more optimal development and growth when compared to no renewal of media during exposure (static method). This could potentially be due to enhanced oxygenation and reduced metabolic waste. Moreover, semi-static conditions may support more consistent test substance concentrations in the media compared to a static method, improving assay sensitivity for morphological malformations following exposure to teratogenic substances [21].

The last parameter that was investigated was the use of a solvent, in this case, 0.5% *v*/*v* DMSO. No obvious differences were seen in either mortality, development, or incidence of malformations between embryos exposed at 0.5% *v*/*v* DMSO and untreated exposure medium, supporting its safe use at this concentration. These results align with those of Selderslaghs et al., who reported adverse effects only at concentrations exceeding 1.5% *v*/*v* DMSO [18]. Nevertheless, it is recommended that solvents are used with caution. The current OECD recommendation is to use no more than 0.01% of solvent [22]. Some authors suggest even lower concentrations, i.e., 0.002% [23]. The latter is also recommended for testing on endocrine disruption, e.g., OECD 231 [24].

Based on the acquired data, and taking into account ethical considerations, it was concluded that the most optimal results in the ZEDTA can be obtained when larvae are incubated in 24-well plates at an incubation temperature of 26 °C with a 48 h medium refreshment interval.

The current study assessed the impact of most proposed conditions of Beekhuijzen et al. [15], with the exception of the impact of chorion removal. In the literature, the effects of chorion removal are debated. On the one hand, the chorion may act as a barrier and could thus limit the exposure to compounds depending on their molecular size and physicochemical properties [4,25]. However, on the other hand, removal of the chorion may increase mortality rates and/or incidence of malformations [26], which may reduce the specificity to detect non-teratogenic substances [12]. Given these drawbacks and the associated labor and costs involved in chorion removal, the experiments in the current study were performed with intact chorions.

In the future, further optimization of the ZEDTA protocol could benefit from incorporating transcriptomic analyses alongside morphological assessments, as suggested by Hamm et al. [27]. Their study highlights how molecular endpoints can detect subtle, mechanism-related developmental effects that may be missed by morphology alone [26]. The absence of transcriptomic analyses in the current study, however, is not considered a limitation, as these endpoints exceed the scope of the routine ZEDTA.

### 4.1. Historical Control Data Derived from the Optimized ZEDTA Protocol [20]

The blank controls of the experiments conducted with the optimized ZEDTA protocol were used for the collection of historical control data to characterize the background mortality rate and the incidence of spontaneous malformations of zebrafish embryos and larvae.

An overall survival rate of 96% (mortality 3.5%) was observed, consistent with results from the optimization experiments. It was noted that the distribution of developmental scores showed a temporal shift: at 48 h post-exposure, over 85% of embryos reached the maximum score, decreasing to over 60% at 96 h. Nonetheless, at all time points, the majority of the observed zebrafish reached the highest or second-highest developmental score, indicating that minor variability exists even within blank control conditions.

With regard to the incidence of malformations, out of the 502 surviving larvae, malformations were noted in 38 larvae (7.6%), of which the majority (68%) had one malformation only. Most frequently occurring malformations included malformations of the yolk sac, tail, and heart, which were noted in 4.0%, 2.8%, and 2.6% of all surviving larvae after 96 h of exposure, respectively. These results complement those of Mori et al. [16], who reported that spontaneous malformations were rarely observed in controls when embryos were carefully pre-selected during early development. Their findings emphasize that the choice of control selection strategy can influence baseline variability. Together, the historical control dataset generated here, and the visual atlas of malformations described by Mori et al. [16], provide complementary resources for interpreting outcomes in future ZEDTA studies.

Overall, these baseline data are valuable for contextualizing results from future ZEDTA screening studies. Moreover, these data can be used to perform a first estimate on the number of embryos needed to screen for a certain test-item effect. Based on Fisher’s exact test and the observed rate of malformations in the control group, groups of 20 embryos are sufficient to detect a 30% increase in malformations. Higher numbers of embryos (e.g., 30–40 embryos) enable detection of smaller effect sizes (15–25%). This can be used as guidance to determine study design decisions for early screening and regulatory application of the ZEDTA. If the ZEDTA is to be implemented to replace rat and rabbit studies for developmental toxicity assessment, future research is still needed to compare study outcomes between both test systems and to assess the number of embryos needed to achieve sufficient power.

### 4.2. Limitations

It should be noted that some experiments of the optimization study were not replicated in triplicate. For example, only two plates were tested for 96-well exposures at both 26 °C and 28 °C, and only one plate was evaluated at 28 °C in 24-well format with 48 h refreshment. Nevertheless, sufficient embryos were analyzed under each condition to draw meaningful conclusions. Specifically, the results from 96-well plates demonstrated clear negative impacts on larval development, and the effects of the single 24-well plate that was incubated at 28 °C with a 48 h semi-static exposure were corroborated by results from other experimental groups, either at 28 °C or with a similar semi-static exposure. While statistical analyses were not performed for all comparisons, the observed trends were consistent and biologically meaningful, providing confidence in the conclusions drawn.

## 5. Conclusions

The most optimal results in the ZEDTA, considering scientific data quality, ethical standards, and cost-effectiveness, can be obtained with an incubation temperature of 26 °C, culturing in 24-well plates, and with media refreshment after 48 h of exposure. Furthermore, our results demonstrate that a concentration of 0.5% *v*/*v* DMSO can be safely used as solvent without negatively affecting the development of the embryos.

The historical control data that were collected in this study under these optimized conditions will be highly valuable in interpreting results from future ZEDTA studies conducted under comparable conditions. Importantly, this study provides the first combined approach of protocol optimization and a comprehensive historical control dataset for both embryonic and early larval stages, representing a novel contribution to the field.

Several next steps can further strengthen the utility and regulatory relevance of the ZEDTA. Inter-laboratory validation of the optimized protocol is essential to confirm reproducibility. Expanding the historical control dataset to include additional strains and larger sample sizes will enhance confidence in background malformation rates and support robust interpretation of chemical effects. And, applying the ZEDTA to a diverse set of chemical classes will help define its predictive domain and translational concordance with in vivo developmental toxicity data.

Ultimately, these developments could facilitate the integration of the ZEDTA into international guideline frameworks, supporting broader acceptance in regulatory toxicology and contributing to the global reduction in animal use in developmental toxicity testing.

## Figures and Tables

**Table 1:** Teratogenic endpoints.

Endpoints Assessed in the Embryo/Larvae	Description
Malformation of the head	Missing structures such as the jaw and the eyes. Uneven eye shape, head shape, edema, etc.
Malformation of the sacculi/otoliths	Missing structures, duplicated structures, or uneven shape.
Malformation of the tail	Kinked tails.
Malformation of the heart	Irregular shape due to edema or aplasia. Abnormal heartbeat.
Malformed body shape	Scoliosis and rachischisis. Also, the notochord presence and its morphology are assessed.
Yolk malformation	Edema.
Pectoral fins	Irregular shape or absence.

**Table 2:** Effects of solvent, temperature, well size, and refreshment interval on larvae survival.

Control Treatment	Temp. (°C)	Well Plate	Refreshment Interval (h)	Eggs per Plate	Total Number of Plates	Total Number of Surviving Larvae	Survival (%)	
							Mean	St Dev
Blank	26	24	24	24	17	386	95	6.7
			48	24	6	138	96	6.5
			96	24	7	159	95	7.9
	26	96	24	20	2	37	93	3.5
	28	24	24	24	10	231	96	5.0
			48	24	1	23	96	n.a.
			96	24	3	72	100	0.0
		96	24	20	2	35	88	3.5
0.5% *v*/*v* DMSO	26	24	24	24	3	67	93	8.7
		96	24	20	3	55	92	7.6
	28	24	24	24	3	67	93	6.4
		96	24	20	3	52	87	2.9

n.a.—not applicable.

**Table 3:** The effects of solvent, temperature, well size, and refreshment interval on the modal General Morphology Scores at different time intervals.

Control Treatment	Temp. (°C)	Well Plate	Refreshment Interval (h)	Eggs per Plate	GMS at Time of Exposure ^1^			
					24 h (7)	48 h (12)	72 h (15)	96 h ^2^ (18)
Blank	26	24	24	24	7	12	15	18
			48	24	7	12	14	18
			96	24	7	12	15	16
	26	96	24	20	7	12	15	17
	28	24	24	24	7	12	15	18
			48 ^3^	24 ^3^	7 ^3^	12 ^3^	15 ^3^	18 ^3^
			96	24	7	12	15	17
		96	24	20	7	12	15	17
0.5% *v*/*v* DMSO	26	24	24	24	7	12	15	18
		96	24	20	7	11	15	18
	28	24	24	24	7	12	15	18
		96	24	20	7	12	15	17

^1^ the number between brackets indicates the maximum possible GMS at the given time point; ^2^ colors in the last column show the gradation of development (green—optimal, orange/red—retarded, in which red refers to a worse condition); ^3^ only one plate was examined.

**Table 4:** The distribution of General Morphology Scores at the end of the exposure (96 h).

Control Treatment	Temp. (°C)	Well Plate	Refreshment Interval (h)	Total Number of Plates	% of Larvae Showing a Given Score		
					16	17	18
Blank	26	24	24	17	0	41	59
			48	6	0	0	100
			96	7	71	29	0
	26	96	24	2	0	100	0
	28	24	24	10	0	20	80
			48	1	0	0	100
			96	3	0	67	33
		96	24	2	0	100	0
0.5% *v*/*v* DMSO	26	24	24	3	0	0	100
		96	24	3	33	33	33
	28	24	24	3	0	0	100
		96	24	3	33	67	0

**Table 5:** Mean larval length (mm) at the end of the exposure (optimization study).

Control Treatment	Temp. (°C)	Well Plate	Refreshment Interval (h)	Average Length (mm) ^1^	Std Dev	Number of Examined Larvae
Blank	26	24	24	3.04	1.08	210
			48	3.66	0.22	30
			96	1.66	0.18	70
		96	24	3.58	0.24	60
	28	24	24	3.49	0.85	160
			48	3.63	0.30	30
			96	1.65	0.17	40
		96	24	3.37	0.27	60

^1^ A color gradient is used to illustrate length distribution, with red indicating the shortest values, orange and yellow representing intermediate lengths and green showing the longest values.

**Table 6:** Overview table on the absolute and relative frequency of specific malformations at the end of the exposure period (96 h).

Control Treatment	Temp. (°C)	Well Plate	Refreshment Interval (h)	Number of Examined Larvae	Percentage of Larvae Showing Given Malformations ^1^						Average Teratogenic Score ^2^
					Head	Sacculi/Otholiths	Tail	Heart	Body Shape	Yolk	
Blank	26	24	24	386	0.52 (2)	0.26 (1)	4.7 (18)	11 (41)	3.9 (15)	4.7 (18)	0.25
			48	138	0.72 (1)	0	1.4 (2)	3.6 (5)	1.4 (2)	20 (27)	0.27
			96	159	0	0	1.3 (2)	20 (32)	3.1 (5)	14 (22)	0.39
	26	96	24	37	0	0	2.7 (1)	41 (15)	5.4 (2)	27 (10)	0.74
	28	24	24	231	1.3 (3)	0.87 (2)	2.6 (6)	9.1 (21)	2.6 (6)	2.6 (6)	0.19
			48	23	0	0	0	8.7 (2)	0	4.3 (1)	0.13
			96	72	0	0	0	8.3 (6)	0	13 (9)	0.21
		96	24	35	2.9 (1)	0	0	14 (5)	5.7 (2)	14 (5)	0.37
0.5% *v*/*v* DMSO	26	24	24	67	1.5 (1)	0	4.5 (3)	4.5 (3)	3.0 (2)	3.0 (2)	0.16
		96	24	55	0	0	5.5 (3)	9.1 (5)	0	5.5 (3)	0.19
	28	24	24	67	0	0	3.0 (2)	3.0 (2)	0	0	0.056
		96	24	52	9.6 (5)	0	3.8 (2)	31 (16)	15 (8)	7.7 (4)	0.68

^1^ between brackets, the number of larvae showing given malformation is presented. ^2^ A color gradient is used to illustrate gradation of teratogenicity, with red indicating the highest scores, orange and yellow representing intermediate scores and green showing the lowest scores.

**Table 7:** Distribution of developmental scores and mortality of embryos/larvae at different time points in the control treatments incubated under optimized conditions.

Hours of Exposure (Maximal Possible Score)	% Zebrafish with a Given Score															Total Examined	Mortality (%)
	2	4	5	6	7	9	10	11	12	13	14	15	16	17	18		
24 (7)	0.21	0.41	0.41	13	86											486 ^1^	2.9
48 (12)		0.20			0.20	0.60	0.20	4.4	94							503	3.3
72 (15)			0.21			0.21			0.21	2.1	40	57				485 ^2^	3.3
96 (18)				0.20				0.20			0.80	1.2	6.6	29	62	502	3.5

The color coding indicates the maximum possible score (dark green) and second-highest possible score (light green) of that respective time point. ^1^ embryos from one plate were not examined at 24 h of exposure. ^2^ larvae from one plate were not examined at 72 h of exposure.

**Table 8:** Cumulative number of malformations observed in larvae after 96 h of exposure in the control treatments incubated under optimized conditions.

Total Number of Malformations per Larvae	Number of Larvae (% of Total)	% of Surviving Larvae
1	26 (68)	5.2
2	5 (13)	1.0
3	3 (7.9)	0.60
4	2 (5.3)	0.40
5	0 (0)	0.0
6	1 (2.6)	0.20
7	1 (2.6)	0.20
Total larvae	38	7.6

**Table 9:** Frequency of malformations in the control treatments incubated under optimized conditions.

Malformation	Number of Malformations (% of Total)	% of Surviving Larvae
Sacculi/otholiths	1 (1.5)	0.20
Body shape	4 (6.1)	0.80
Pectoral fins	6 (9.1)	1.20
Head	8 (12)	1.59
Heart	13 (20)	2.59
Tail	14 (21)	2.79
Yolk	20 (30)	3.98

## Data Availability

The original contributions presented in this study are included in the article. Further inquiries can be directed to the corresponding author.

## Glossary

Egg	Fertilized egg including embryo, perivitelline space and chorion.
Embryo	Organism before hatching.
Larvae	Organism after hatching.
Static exposure	No renewal of exposure medium during the entire exposure period
Semi-static exposure	Renewal of exposure medium at defined intervals.
Malformation	A structural defect in the body due to abnormal embryonic or larval development.
Dead embryo (coagulated)	Macroscopic appearance on dark underground is white, and microscopic appearance is brownish, hampering the transparency of the embryo. This is accompanied by lack of heartbeat when observed at 48 h post-fertilization and later.
Dead larvae	Immobility and/or absence of respiratory movement and/or absence of a heartbeat and/or white opaque coloration of the central nervous system and/or lack of reaction to mechanical stimulus.
